# Supplementary material for: DMRforPairs: identifying Differentially Methylated Regions between unique samples using array based methylation profiles
Source: BMC Bioinformatics. 2014 May 15;15:141. doi: 10.1186/1471-2105-15-141 (PMC4046028; doi:10.1186/1471-2105-15-141)
Supplement: Additional file 2 — DMRforPairs output for the comparison of A431-MCF7 and NA17018-NA17105. Please start from the HTML files in each folder. Available via the BMC Bioinformatics website. [file 1471-2105-15-141-S2.zip › 1394847754114233_MOESM2_ESM/A431_MCF7/figures/34363.pdf]

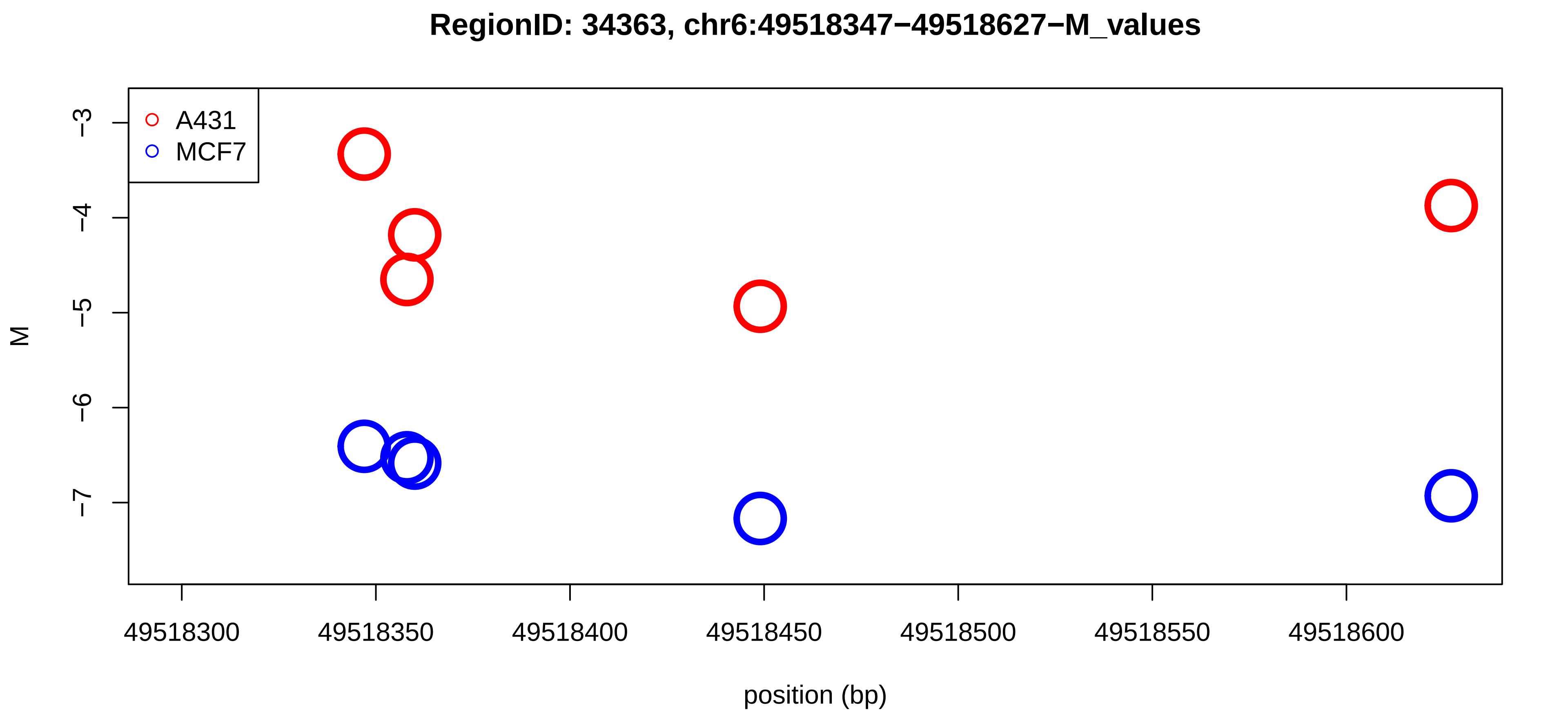

RegionID: 34363, chr6:49518347−49518627−M_values
A431
MCF7
M
−3
−4
−5
−6
−7
49518300
49518350
49518400
49518450
49518500
49518550
49518600
position (bp)

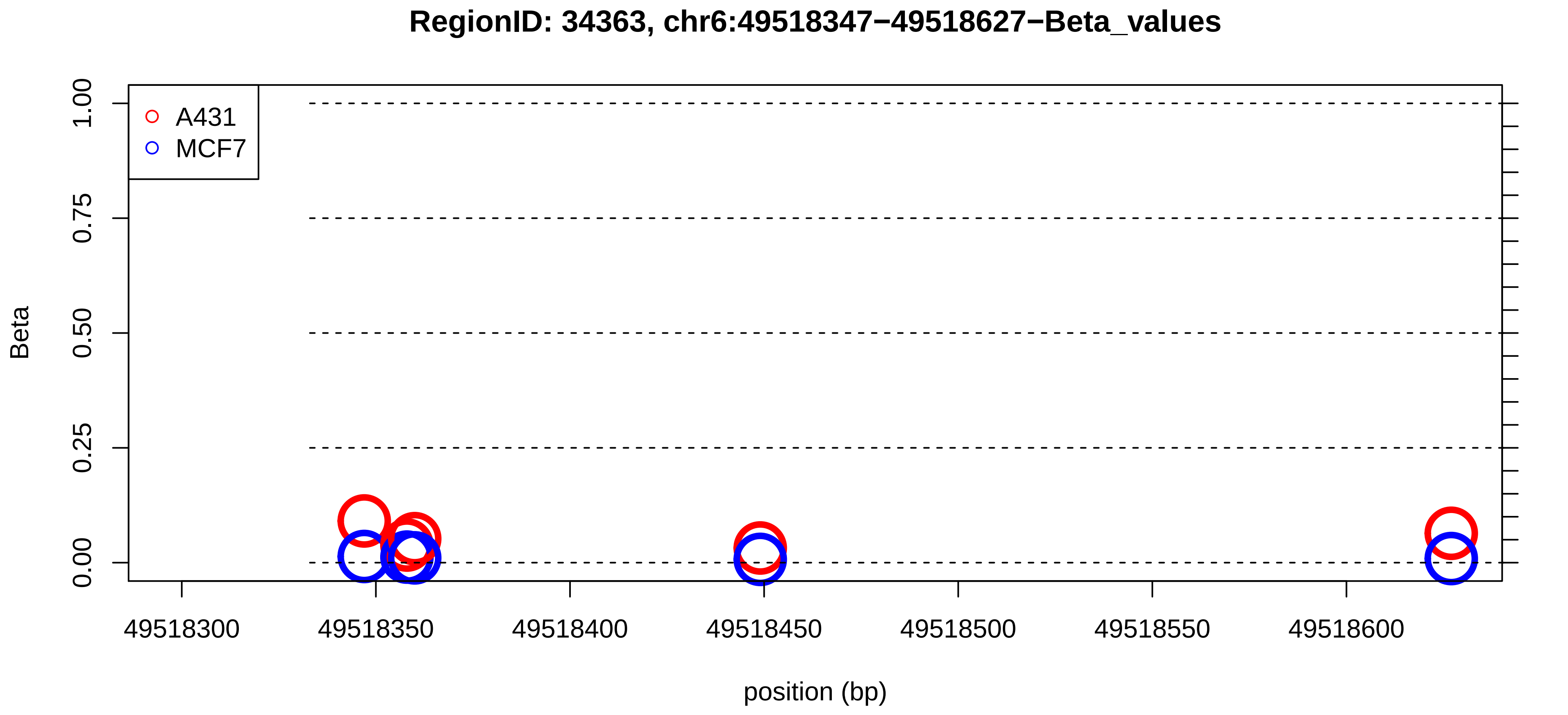

RegionID: 34363, chr6:49518347−49518627−Beta_values
A431
MCF7
Beta
1.00
0.75
0.50
0.25
0.00
49518300
49518350
49518400
49518450
49518500
49518550
49518600
position (bp)

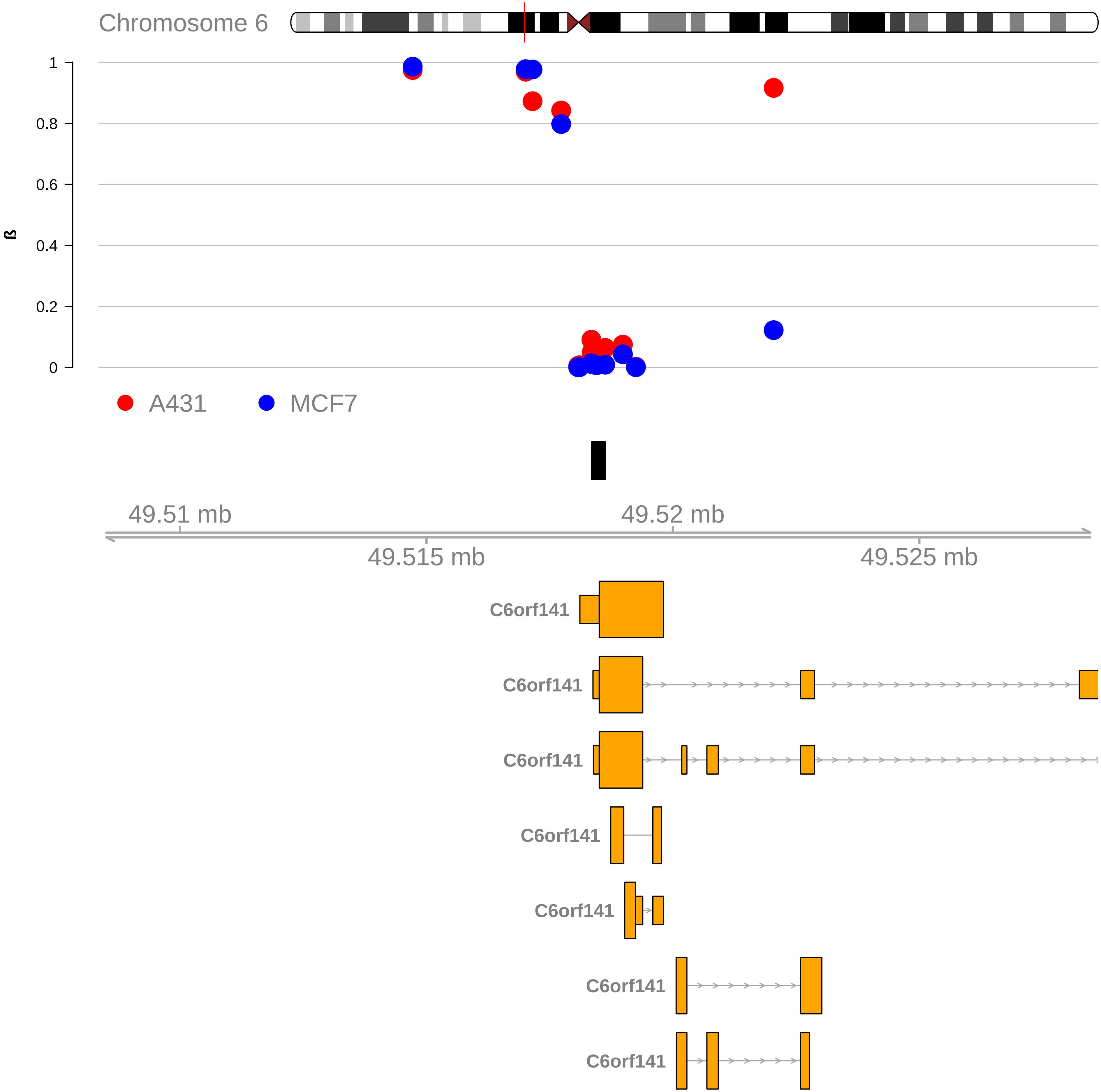
Chromosome 6
1
0.8
0.6
0.4
0.2
0
ß
A431
MCF7
49.51 mb
49.52 mb
49.515 mb
49.525 mb
C6orf141
C6orf141
C6orf141
C6orf141
C6orf141
C6orf141
C6orf141
